# Adolescents with congenital heart disease: their opinions about the preparation for transfer to adult care

**DOI:** 10.1007/s00431-017-2917-9

**Published:** 2017-05-16

**Authors:** Åsa Burström, Ewa-Lena Bratt, Björn Frenckner, Margret Nisell, Katarina Hanséus, Annika Rydberg, Maria Öjmyr-Joelsson

**Affiliations:** 1 Institution for Women’s and Children’s Health, Karolinska Institutet, Stockholm, Sweden; 2 Department of Paediatric Cardiology, Astrid Lindgren Children’s Hospital, Stockholm, Sweden; 3 Institution of Health and Care Sciences, University of Gothenburg, Gothenburg, Sweden; 4 Department of Pediatric Cardiology, The Queen Silvia Children’s Hospital, Gothenburg, Sweden; 5 Department of Pediatric Cardiology, Skåne University Hospital, Lund, Sweden; 6 The Red Cross University College, Stockholm, Sweden; 7 Department of Clinical Sciences, Pediatrics, Umeå University, Umeå, Sweden

**Keywords:** Congenital heart disease, Transition, Adolescents, Focus group interviews

## Abstract

The aim of the study was to explore what adolescents with congenital heart disease (CHD) view as important in the preparation for the transfer to adult care. We performed interviews in four focus groups with adolescents (14–18 years old) at four university hospitals in Sweden. Data was analysed using qualitative content analysis. The analysis revealed one main category; *Becoming a manager of the condition* and four subcategories; *Sufficient knowledge about the health, Be a participant in the care, Parental support,* and *Communicate with others about the health.* The adolescents’ ages differentiated the discussion in the groups. The older adolescents seemed to have more interest in transition planning, information and transfer. The younger described more frustrations about communication and handling the disease.

*Conclusion*: To become a manager of the CHD in daily life, the adolescents want disease specific knowledge, which should be communicated in a developmentally appropriate way. Adolescents want to participate and be involved in the transition process. They need support and guidance in how to communicate their CHD. Parental support is fundamental but it change over time. Moreover, peer-support is becoming more significant during the transition process.

**Table:** 

**What is Known:** *• Transition during adolescence and transfer to adult care for adolescents with CHD is complex, and there is a shift in roles.* *• Adolescents often have poor knowledge and understanding about their heart condition and the consequences.*
**What is New:** *• Adolescents call for disease specific information regarding health issues of importance for them in daily life.* *• Communicating the disease with other is a challenge- peer support from other adolescents with CHD could be a facilitator.*

## Introduction

For the majority of children born with congenital heart disease (CHD), the medical and surgical progress has resulted in an increased life expectancy [15, 16, 22, 30, 53, 55]. To maximize the potential and lifetime functioning for this growing group of adolescents, lifelong care is needed according to European, American and Canadian guidelines [1, 49]. In order to ensure a lifetime follow-up, the paediatric-to-adult transfer of care should be preceded by a preparatory transitional phase for the adolescents [20, 39].

During the transition, young people need to learn about their health and heart conditions and gradually take over responsibility for their healthcare. By adopting good health behaviour, the risk for late complications can decrease [23]. However, this transition is also a part of a wider general developmental transition process for the young person, including puberty, which is accompanied by physical, psychological, and emotional changes [35]. In addition, the young persons’ identity-seeking is evolving and they are beginning to make education and occupation choices [3, 60].

Furthermore, several studies indicate that adolescents do not feel included and involved in the preparation before the transfer [13, 57, 58, 63], which also may increase the risk of loss of follow-up after transfer to adult care [6].

In Sweden, there are no consensus guidelines on how young persons with long-term medical diseases such as CHD should be prepared during the transition prior to the transfer. Routines differ between hospitals and are also person-dependent. In some settings, the young person >12 years or older is offered an individual meeting with the healthcare providers (HCPs)—the physician and the nurse—(without the parents). But this is not mandatory. In some CHD-centres, young persons are introduced to the adult care giver and setting before the transfer to the adult care unit, grown-up congenital heart disease-clinic (GUCH). Most of the information during the transition, about the transition and transfer process is given by a paediatric cardiologist, sometimes in collaboration with a nurse.

## Objective

The aim of this study was to explore what adolescents with CHD view as important in the preparation for transfer to adult care.

## Methods

### Setting and participants

The participants were recruited from four paediatric cardiology centres at university hospitals in Sweden. Purposive sampling was applied. The inclusion criteria were adolescents with moderate to complex CHD [49, 62], age between 14 and 18 years old, Swedish-speaking and being able to participate in a group discussion. At each participating centre, a nurse or physician was asked to select six to eight adolescents with equal gender distribution that met the inclusion criteria. Information letters describing the focus group interview and consent forms were sent to each of the prospective informants, as well as to their guardians because the adolescents were younger than 18 years old.

### Data collection

Focus group interviews were used for data collection. The method was chosen to explore the adolescents’ opinion in relation to different centres and ages. In focus groups, data is produced by the participants’ opinions and interactions with other participants in the group [32]. The interviews were audio-recorded and lead by the first author (ÅB). The co-author (ELB) took notes about the interaction, seeing to it that all adolescents were included. The interviews were performed in the late afternoon in a room at each hospital separated from the outpatient clinic. An interview guide had been developed by the research team and was used during the interviews (Table 1). Each interview lasted 50–65 min. Before starting all interviews, confidentiality was explained to ensure that every participant understood the meaning and felt they could talk freely. All participants were asked to introduce themselves, but not give their diagnosis or age, as adolescents can be very age-conscious [25].

**Table 1:** Interview guide

• Can you tell me what you think is important for adolescents to know before they leave the paediatric cardiology department? • What do you think is important to know about the heart condition?, i.e. complications, follow-up, education, medical treatment, lifestyle issues, physical activities, contraceptives and family planning. • What do you think about parents’ need for preparation? • What do you think is the most important before transfer to adult care? • When do you consider the optimal time for transition preparation to start? • When do you consider the optimal time for the transfer to take place? • Do you think that it is age that should be the deciding factor? • How do you think the information about transition and transfer should be given? • Have you heard about GUCH? • Is there anything you consider important that we have not discussed today?	

### Data analysis

The interviews were transcribed verbatim and analysed using content analysis with a manifest approach [25]. Each group interview was analysed as one text unit. The text was read several times in order to get a sense of the whole. Then open coding was conducted on each text unit. The codes from the four text units were compared and contrasted to find similarities and differences between the groups, but also within each group [25]. The codes were grouped and formed categories. The main category included and described the meaning of the four subcategories. The analysis was made together with the co-researchers (ELB, MÖJ and MN) to achieve trustworthiness. Additionally, quotations from dialogues in the groups were used for illustrating the categories [25].

## Results

In total, 19 adolescents accepted to participate but due to subsequent dropouts, the number ended up to 17 (Table 2). Four focus group interviews were performed, including three to five adolescents in each group. The analysis from the interviews revealed one main category, *Becoming a manager of the condition* and four subcategories, including *Sufficient knowledge about the health, Be a participant in the care, Parental support* and *Communicating with others about the health*.

**Table 2:** Demographic and clinical characteristics of the study population (n = 17)

Gender	
Female	10
Male	7
Age	
14	5
15	4
16	2
17	2
18	4
Mean age (SD +/-)	15.8 (1.6)
Lesions	
Moderate grade^a^	2
Complex grade^b^	15

^a^Aortic stenosis, pulmonary stenosis

^b^Univentricular, transposition of the great arteries (TGA), double outlet right ventricle (Dorv) and tetralogy of fallot (ToF), congenitally corrected transposition (CcTga) and isomerism

The adolescents’ ages differentiated the discussion in the groups. The older adolescents seemed to have more interest in transition planning, information and transfer. The younger participants in general described more frustrations about communication and handling their disease. Another difference about the future transfer was that it was viewed differently depending on the participants’ age. For the older adolescents, a future transfer to adult healthcare was a natural step, but many of the younger participants had never thought about it.

### Sufficient knowledge about the health

Trust for the HCPs’ experience and knowledge was evident in all groups. Information given to them about their CHD and health-related information was considered as important—not only how it was given, but also the content of the information was crucial. The paediatric cardiologist gave information about the CHD, health-related information and treatment during the medical check-up at the outpatient clinic. The adolescents said that they had knowledge about their diagnoses and medication. However, sometimes it was difficult to understand and remember the information given by the physician. Some of the adolescents with pharmacological treatment missed information about their treatment, about risks of not taking the prescribed medication and also about side effects.

All groups discussed the deficiency of explicit information about issues that might have an impact on their health including alcohol, smoking, piercing, physical activities and physical restrictions. There were differences in what information they received about piercing and tattoos, which was found annoying. They described the information as inaccurate. In one group, questions about contraceptives and pregnancies were discussed among the older participants (boy 17 years and girl 18 years). Some of the adolescents received instructions to participate in sport class but to avoid being exhausted. This kind of information was difficult and contradictory to understand. The topics resulted in long discussions. They asked for more accurate and specific explanations based on the individual medical situations. A dialogue from one of the groups about difficulties to understand given information:

I’ve been told not to get too exhausted in sport class and so on... and that I must tell the teacher when or if that happens… but no one has ever told me why…// … it is difficult to understand the balance… Am I tired because of my heart condition or is it because I need to exercise more… (Girl 14)

Yes, you might wonder what should you be aware of…? (Boy 17)

Exactly! That is difficult to know. Am I tired just like everybody else or is it because of my condition…? (Girl 18)

Information about drugs and smoking was discussed in all groups and was considered as important knowledge. The adolescents said; “If the heart is weaker, one should know what smoking and alcohol is doing to the heart”. It was important to understand the impact and the consequences. However, sometimes they also found the information exaggerated. A dialogue from another group:

I must say, about the consequences... oh my god how they spoke about drinking and sex at my last visit! - If you drink alcohol you’re going to die or end up in an emergency room. You are not allowed to get drunk… and if you have unprotected sex you’re going to die”… oh my god… it was like they had to tell me everything, it was horrible! But hallo, I’m not stupid…it was too much, I was devastated… You have to individualize such information…! // You can start giving this information during youth, but not too early… (Girl 18)

But you can start at 15, to just talk about it, but not like her physician did… I was told that I could drink alcohol, but not on a regular basis or too much. (Boy 18)

Oh god so boring (laugh)… I’m sorry, no but of course, you should inform the patients but not frighten them… I was afraid to die if I tried (Girl 18)

I agree, you can tell it in a more gentle way…// But for me it is not an issue, I see it as a gain… I don’t need to drink alcohol (Boy 18)

### Be a participant in the care

Learning to administer their medication was a way to increase the responsibility of the adolescents’ self-care. It came naturally for some adolescents, i.e. when they started sleeping over with friends. Difficult names of the medications but also how difficult it could be to remember to administer the pills was also discussed. A dialogue between two girls who share the common experience:

I have real difficulties remembering to take my medication…I don’t know why... don’t know. I really try. (Girl 14)

Me too…must say... forget it all the time. Use the alarm on your cell phone! I do it all the time! Or use notes or maps, day by day. It helps to see if or what you have forgotten! (Girl 18)

To increase their involvement in the care, the adolescents in all groups wanted their physician to address the questions to them and not to the parents. This could be supportive and help them to be more reflective about themselves and their healthcare management. However, older participants expressed that it was important that the meeting with the physician was a dialogue and not just a monologue with questions directed to the adolescent. They wanted information about the transfer 2 or 3 years ahead. The majority of the participants, except for a few of the youngest, expressed the importance of being introduced to the adult care setting before the transfer. They understood the need to continue the medical follow-up as adults, but in one group, they described the information as implied and unclear. One girl described how the HCPs began to talk about the adult care clinic, GUCH, at the last visit. She felt unprepared and it was unexpected, while the concept, GUCH, was something she never had heard about. It was confirmed in the other groups. Only one adolescent in all groups had ever heard about GUCH. The groups seemed not to have understood that GUCH was the name of the adult clinic. A quote from one group:

GUCH here and GUCH there… but what is that. Is it a kind of organisation I need? I don’t know… (Girl 18)

### Parental support

Parental support through the transition process was important in several ways. Several participants in the groups described how parents supported them before hospital visits by asking them t*o* write notes of potential questions or help by helping them by reminding at the appointment; “You had a question about…”.

The parents were also considered important in recalling the information given. To meet the physician alone during the appointment was discussed in all groups. For some of the younger participants, it was important to have the parents nearby if they felt shy and had difficulties to expressing themselves. This, though, was something that changed during adolescence and varied based on maturity. For most of the older adolescents, it was important to have the opportunity to have a private talk with the physician; however, it was described as comforting to have the parents waiting in an adjacent room during the physicians’ appointments. Even though the importance of having a meeting without the parents present was emphasized, as it had to be introduced in advance to prepare the parents and adolescents. Here is a dialogue from one of the groups about to meet the physician unprepared and without the parents:

I felt forced the first time they said that I must go alone without my parents. I refused because I wanted to have my parents with me. // You don’t know so much about life when you are 13 (Girl 18)

No, you don’t have the whole picture…. (Boy 18)

Hmm I thought it was tough to meet the doctor alone …(Girl 14)

I didn’t find it so tough, but I wondered why do I had to come alone…

(Girl 15)

### Communicating with others about the condition

Learning to communicate their condition was sometimes described as challenging. In two of the groups, the adolescents explicitly expressed that it was helpful to have an opportunity to talk with peers in the same situation. In one group, they discussed the difficulties of explaining for others about their CHD, for instance when they needed to take a break in sport class or when classmates asked questions about their scars. Although their school were informed about their extra need of rest, they found it annoying when teachers or others seemed worried and they could not explain themselves. A dialogue from one of the groups:

It feels like, every time I lie down on the floor to rest…(Girl 14)

…They think you’re lazy or dead (Girl 18)

(Laughter)… Yes, exactly… just because you have a heart condition (Girl 14*)*

Yes, it is hilarious… (Girl 18)

They also discussed how to handle questions about scars. Some accepted it while others described themselves as shy and with an uncomfortable feeling of being different. One of the younger participants got annoyed when people asked about the scar, while others who were older told about how to make up stories. One of the older participants spoke about how the way she handled people’s questions had changed over the years. Nowadays, she could feel more confident about it.

### Interaction in the groups

The atmosphere in all of the focus groups was friendly, and all adolescents participated in the discussion and shared their opinions with each other. However, the older participants were more active and communicative, but they invited the younger participants into the discussions by allowing them to express their opinions, filling in and describing similar experiences from when they were younger. Furthermore, the differences within the groups were similar according to age and maturity. The older adolescents were more prone to have an interest in talking about the transfer process, and the youngest participants described difficulties in communicating about their health with others. The following quote is a dialogue between a girl 18 years and a boy 14 years:

I’ll have my last appointment on Friday,…it makes me wonder… I don’t know, what will be the difference? (Girl 18)

I have never thought about it…(Boy 14)

## Discussion

This study provides information about what aspects adolescents with CHD consider important during the transition, in order to get prepared and gain control of their health. It also shows how the HCPs need to be aware of the progress of the maturation process [35] during the transition in order to meet the adolescents’ needs. HCPs have to take the adolescents’ bio-psychosocial developmental needs into consideration as it may affect the care [12].

Sufficient knowledge about the health situation is an important aspect for a successful transfer [17, 31], and for paediatric cardiologists and nurses, it is an obligation and a challenge to meet the adolescents’ need for knowledge and preparation [39, 42]. The adolescents in this study expressed difficulties in understanding information given in several areas related to their health. They reported that the information sometimes could be insufficient and that there was not enough of a dialogue. This might be important to remember when giving health-related information, as dialogues have been recognized to be more effective for the purpose [38]. Furthermore, information given about the CHD and health-related information can be difficult for the adolescence to understand. During adolescence and puberty and into their early twenties, the adolescents are developing an ability to think abstractly and concretely and to understand long-term consequences [8]. In addition, there might be a discrepancy in what they consider important to learn more about and what the HCPs consider important when it comes to health-related information. The adolescents seem to be more “in the here and now” of what is interesting to learn about [7, 59] issues like piercing, while HCPs are more into-long term consequences as in our study. It emphasizes the importance of ensuring that information is understood [26]. Asking the adolescent to give a short summary in the end of the meeting can be one way to establish that. Inaccurate knowledge about the CHD might lead to unnecessary restrictions [48], avoidance of deeper relations [6] and uncertainty [2, 4, 5, 22]. The majority of the adolescents in our study said that they had general knowledge about their CHD; something they learned over the years in the paediatric setting. However, they expressed a need for more knowledge about health related topics. Other studies show similar results—that most of the adolescents with CHD have at least basic knowledge about their condition but that they show gaps and needing more information in other subjects such as contraceptives, pregnancies, drugs and occupational choices [9, 14]. Contraceptives and future pregnancies were only discussed in one of the groups and between the older participants. One reason might be that sensitive topics can be difficult to discuss in a group, due to levels of maturity and different levels of significance for the adolescents [7, 10, 59]. According to the group in the present study who discussed it, this topic had not been sufficiently communicated, which is consistent with other studies [11, 29, 37, 40, 42]. It is an important topic that may need more attention in health-related information for adolescents with CHD, as pregnancies might have a serious impact on health for women with complex CHD and appropriate contraceptive counselling by a specialist might needed [1, 15, 19, 50, 55].

Information about GUCH and meeting the new adult health care team was one of the most important aspects for adolescents in this study, and it was more prominent among the older participants, which is consistent with Rutishauser’s findings [41]. This was possibly because especially the older adolescents realised that the transfer was not far ahead. By the same token, it was something the younger adolescents seldom thought about. It was considered important to feel included and part of the process for the adolescents. This is helpful for supporting the adolescents during the preparation prior to transfer [18, 41]. Furthermore, adolescents with higher perceived self-efficacy and transfer knowledge are associated with better QoL [54].

Adolescence is a vulnerable time when adolescents are striving for independence and autonomy from their parents at the same time, as they need parental support and guidance [3, 61]. The adolescents in this study revealed the importance of parental support to feel safe even if their needs changed with age. This is important for HCPs to consider, to support and encourage the parents during this phase. When the families do well, the children do it as well [27], and according to Viner et al., supportive parents are one of the most important determinants of health [61]. During the transition and when the adolescents learn to increase involvement and responsibility in their own care, parents need to learn and be supported in their change of roles from managers to be consultants, in order to facilitate their child’s move from dependence to independence [21].

The present study displayed unstructured transition preparations. Some of the participants described how they had felt forced when they were to meet the physician individually for the first time, without their parents beside them. They felt unprepared and too young when they were around 12–13 years old. The procedure was not introduced to them or the parents beforehand and that raised insecurity. This phenomenon is congruent with a Swedish study by Asp [2, 4, 5, 22]. Transition activities, i.e. individual meetings with the physician or transfer need to be announced and to be prepared in advanced in order for the participants to understand the procedure [9, 43–45, 56]. Still, it is known that by inviting adolescents to talk and ask questions without their parents being present is one way to improve autonomy [20]. According to Swedish law, young people have the right to be offered an individual meeting to be informed and discuss their care [52].

The younger participants in this study described it as difficult to express their conditions or needs for rest in school, i.e. after sport activity. It is a delicate issue for the adolescents living with physical limitations and their desire not to stand out, to avoid the risk of being different and thus being stigmatized [28, 51]. The adolescents could not understand the concern the teacher expressed when they rested on the floor after sport activities. Explanations might be the lack of maturity, an egocentric and concrete thinking [7], or a lack of knowledge about the CHD [9]. However, for the younger adolescents it may be difficult to cope with, which might be important for the HCPs to understand.

A successful transition for adolescents born with CHD, with preparation and education prior to the transfer, has been emphasized in previous studies [8, 11, 39, 41, 42] .To prevent damaging health behaviour and enhance knowledge of the CHD, health-related knowledge and the process of a transition program before transfer to adult healthcare may help young people with CHD by enhancing autonomy and facilitating independence [39].

In future perspectives, HCPs should initiate the transition process along with the adolescents and be aware of the importance of parental support. The importance of peer support in addition to parental support has earlier been described by Oris et al. [34], and this is in line with the findings in the present study. Some adolescents seemed to be unaware of the transition process and the future adult caregiver. Further, it seems to be discrepancies about the content in the information given by HCP such as, risky health behaviour (i.e. alcohol, tattoos, etc). There are recommendations for transition coordinators to facilitate and ensure that the adolescents are included and part of the process [24, 33, 42–47, 56] and also consider age and maturity [39]. Hence, there is a need to ensure a structured transition process, and that given information is concordant, this could be addressed and accomplished by a transition coordinator.

## Methodological considerations

Focus group interviews were chosen to collect adolescents’ different opinions about important factors facilitating transition and transfer to GUCH. The method was considered suitable to use as the topic was not considered too sensitive to be discussed in a group [36]. It could also show differences in opinion and topics of interest related to age, which were displayed in the discussions and interactions. An effort was made when introducing all groups to the aim of the group discussion, that it was to share experiences and to discuss opinions about important factors during the transition to transfer. The adolescents were informed that there were no right answers, in order to encourage them not to be shy to talk in the group or be wary of bragging [10]. The focus of the interviews was not to reach consensus but to better understand adolescents with CHD and their different needs and opinions [25]. There might be risks for conformity in a group interview [32, 36]; however, all perspectives were encouraged. A weakness of the study was the low number of participants in the groups; however, the focus group with few participants yielded rich data. Neither did the severity of lesion or gender affected participation in the discussion. It seemed that age and maturity were the important factors for participation in the group discussions.

## Conclusion

To become a manager of the CHD in daily life, the adolescents want disease specific knowledge, which should be communicated in a developmentally appropriate way. Adolescents want to participate and be involved in the transition process. They need support and guidance in how to communicate their CHD. Parental support is fundamental but it change over time. Moreover, peer-support is becoming more significant during the transition process.

## Abbreviations

CHD: Congenital heart disease
GUCH: Grown up with congenital heart disease
HCP: Health care provider
